# Skin sloughing in susceptible and resistant amphibians regulates infection with a fungal pathogen

**DOI:** 10.1038/s41598-017-03605-z

**Published:** 2017-06-14

**Authors:** Michel E. B. Ohmer, Rebecca L. Cramp, Catherine J. M. Russo, Craig R. White, Craig E. Franklin

**Affiliations:** 1 The University of Queensland, School of Biological Sciences, St. Lucia, QLD 4072 Australia; 2 University of Pittsburgh, Department of Biological Sciences, Pittsburgh, PA 15260 United States; 3 Monash University, Centre for Geometric Biology, School of Biological Sciences, Monash, VIC 3800 Australia

## Abstract

The fungal pathogen *Batrachochytrium dendrobatidis* (*Bd*) has been implicated in amphibian population declines globally. Given that *Bd* infection is limited to the skin in post-metamorphic amphibians, routine skin sloughing may regulate infection. Skin sloughing has been shown to reduce the number of cultivatable microbes on amphibian skin, and *Bd* infection increases skin sloughing rates at high loads. However, it is unclear whether species specific differences in skin sloughing patterns could regulate *Bd* population growth on the skin, and influence subsequent infection dynamics. We exposed five Australian frog species to *Bd*, and monitored sloughing rates and infection loads over time. Sloughing reduced *Bd* load on the ventral skin surface, in all five species, despite wide variation in susceptibility to disease. In the least susceptible species, an increase in sloughing rate occurred at lower infection loads, and sloughing reduced *Bd* load up to 100%, leading to infection clearance. Conversely, the drop in *Bd* load with sloughing was only temporary in the more susceptible species. These findings indicate that the ability of sloughing to act as an effective immune defence is species specific, and they have implications for understanding the pattern of *Bd* population growth on individual hosts, as well as population-level effects.

## Introduction

Disease is increasingly recognized as a major threat to wildlife populations^1–3^. Amphibian populations are experiencing declines and extinctions on a global scale^4, 5^, and many of these declines have been attributed to the fungal pathogen *Batrachochytrium dendrobatidis* (*Bd*), which infects the keratinized layers of an amphibian’s skin^6, 7^. Given the importance of the amphibian epidermis for a multitude of physiological functions, from cutaneous gas exchange, to water and ion balance^8^, infection with *Bd* can cause severe disease, known as chytridiomycosis, and mortality^6^. In the post-metamorphic amphibian host, *Bd* is entirely restricted to the skin, and skin defences play a central role in preventing disease^9^. Thus, a better understanding of the physiology of amphibian skin will help elucidate how *Bd* interacts with its amphibian hosts, and particularly, how that interaction varies across species.

Amphibian skin is the first line of defence against invading pathogens, and it is constantly renewed to ensure optimal physiological function^10^. This renewal process is termed sloughing or moulting, in which an amphibian sheds the outer skin layer, the *stratum corneum*, in its entirety on a regular basis^11^. Importantly, amphibian skin shedding has been hypothesized to play a role in the regulation of cutaneous microbes, by periodically removing resident populations of bacteria and fungi^12, 13^. This regulation has been demonstrated to remove up to 100% of the cultivatable microbes on the skin surface^13, 14^, and may help prevent dysbiosis events^15^. Sloughing of the outer layers of skin or scales has been hypothesized to play a role in parasite removal in other species as well, including snakes infected with the fungal pathogen *Ophiodimyces* ^16^ and fish infected with *Gyrodactylus* parasites^17^. Previously, we endeavoured to determine the effect of *Bd* infection on skin sloughing in the Australian green tree frog (*Litoria caerulea*), a species susceptible to *Bd* infection and chytridiomycosis^18^. While high *Bd* infection loads were correlated with increased sloughing rates in individual frogs, *Bd* load on the ventral skin surface did not appear to decrease with sloughing^18^. Based on these initial findings, we hypothesized that sloughing was not effective at removing *Bd* in the susceptible species *L. caerulea*, and that perhaps an increase in sloughing rate may actually exacerbate the loss of physiological homeostasis seen in terminally ill frogs^19, 20^. However, the growth pattern of *Bd* in amphibian skin is host-dependent^21^, and an investigation of sloughing across amphibian species may highlight differences in the role of sloughing in pathogen removal.

*Bd* infects the outer keratinized layers of amphibian epidermis, principally the *stratum corneum* and the *stratum granulosum*, via a swimming flagellated zoospore^6, 22, 23^. After encysting on the surface of the *stratum corneum*, zoospores develop into zoosporangia, and produce germ tubes to invade the deeper skin layers and grow intracellularly^21, 24^. Amphibians have a diverse suite of skin-associated immune defences, such as antimicrobial peptides, which have been shown to correlate with resistance to *Bd* infection in some species^25^, and likely inhibit the growth of *Bd* on the skin^9^. In addition, it has been demonstrated that *Bd* invokes two very different growth patterns in skin explants of susceptible versus tolerant hosts. In susceptible species, such as *L. caeruela* and *Alytes muletensis*, *Bd* is found growing almost exclusively intracellularly in the keratinized and partially-keratinized skin layers^21^. However, in species that demonstrate higher levels of infection tolerance, and eventually infection clearance, such as *Xenopus laevis*, *Bd* population growth is seen as entirely epibiotic, with growth only occurring on the skin surface^21^. This evidence of host-dependent variation in *Bd* population growth pattern may indicate that routine sloughing of the outer *stratum corneum* may be more effective at removing zoospores and zoosporangia in less susceptible amphibian species, in which primarily epibiotic *Bd* population growth occurs.

We set out to determine the role of amphibian skin sloughing in the regulation of *Bd* population growth in five Australian frog species, in order to compare species with different inherent susceptibility to disease. We hypothesized that frog species that experience low mortality rates and infection clearance would demonstrate a decrease in *Bd* load on the ventral skin surface after sloughing. In addition, we investigated whether sloughing rates increase in less susceptible frog species after *Bd* exposure, and if this occurred at lower *Bd* loads. We hypothesized that less susceptible species would increase their sloughing rates at lower infection loads, potentially demonstrating a defensive response to infection. The amphibian integument varies widely in structure and function across species. Understanding the role of the ubiquitous and largely understudied process of amphibian skin sloughing, and how it varies across species, may provide clues as to how some amphibians overcome *Bd* infection. In addition, if sloughing regulates *Bd* population growth on the skin of some frog species, sloughing rate may inform models needed to predict the effects of *Bd* infection at specific sites and populations^26^.

## Results

### Experimental exposure

**Figure 1:**
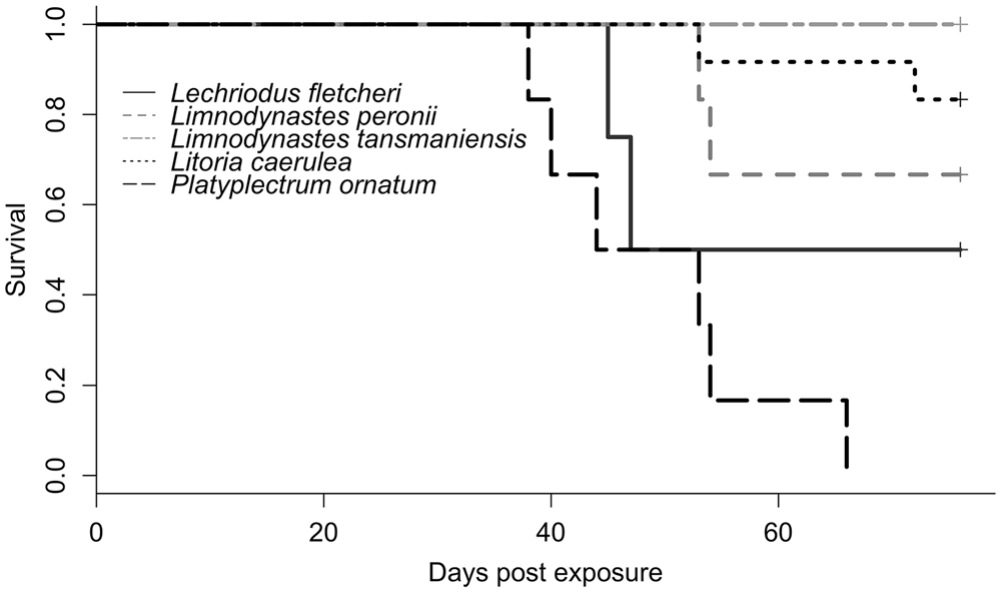
Survival curves for five frog species found in southeastern Queensland, Australia, after exposure to *Batrachochytrium dendrobatidis* (*Bd*).

All control frogs remained *Bd* negative for the duration of the experimental period. Infection prevalence and mortality rate in exposed frogs varied across the five species studied, with *Platyplectrum ornatum* experiencing the highest mortality rate (100%) and average prevalence (73.3%, s.e. = 6.1%), and *Limnodynastes tasmaniensis* experiencing the lowest mortality rate (0%), and average prevalence (34.7%, s.e. = 9.3%; for details for all species see Supplementary Table S2). Survival curves were significantly different between species (χ^2^ = 27.9, d.f. = 4, *p* = 1.29 × 10^−5^; Fig. 1). Both days post exposure and the interaction between days post exposure and species were significant in the model describing infection load over time, which reflects differences in infection outcome between species (e.g. *Bd* clearance or mortality; days post exposure: F = 30.31, d.f. = 1,187.4, *p* = 6.6 × 10^−9^, days post exposure*species: F = 17.85, d.f. = 4, 201.9, *p* = 1.5 × 10^−12^, days post exposure^2^: F = 25.0, d.f. = 1,187.9, *p* = 4.9 × 10^−3^; Supplementary Fig. S2, Table S3). In order to compare sloughing duration based on infection outcome, exposed frogs were divided into the following groups: clinical (developed clinical signs of disease), infected (tested positive for *Bd* without developing clinical signs), and uninfected (never tested positive for *Bd*).

### Sloughing behaviour

In control frogs, *Litoria caerulea* demonstrated the longest intermoult interval (IMI) on average of 4.02 days (±0.47 s.d.), while *Lim. peronii* and *Lim. tasmaniensis* demonstrated the shortest IMIs of 2.47 (±0.53 s.d.) and 2.57 days (±0.49 s.d.), respectively. *Lechriodus fletcheri* and *P. ornatum* sloughed on average every ~3 days (2.82 ± 0.55 s.d. and 2.96 ± 0.41 s.d.). Sloughing behaviour was similar across species and is in line with previous reports^18^, and the duration of the sloughing behaviour did not vary across group (F = 1.34, d.f. = 3, 41, *p* = 0.28), or days post exposure (F = 1.53, d.f. = 1, 619, *p* = 0.22), but did vary across species (χ^2^ = 11.82, d.f. = 1, p = 0.00059; Supplementary Table S4).

### Change in infection load with sloughing

**Figure 2:**
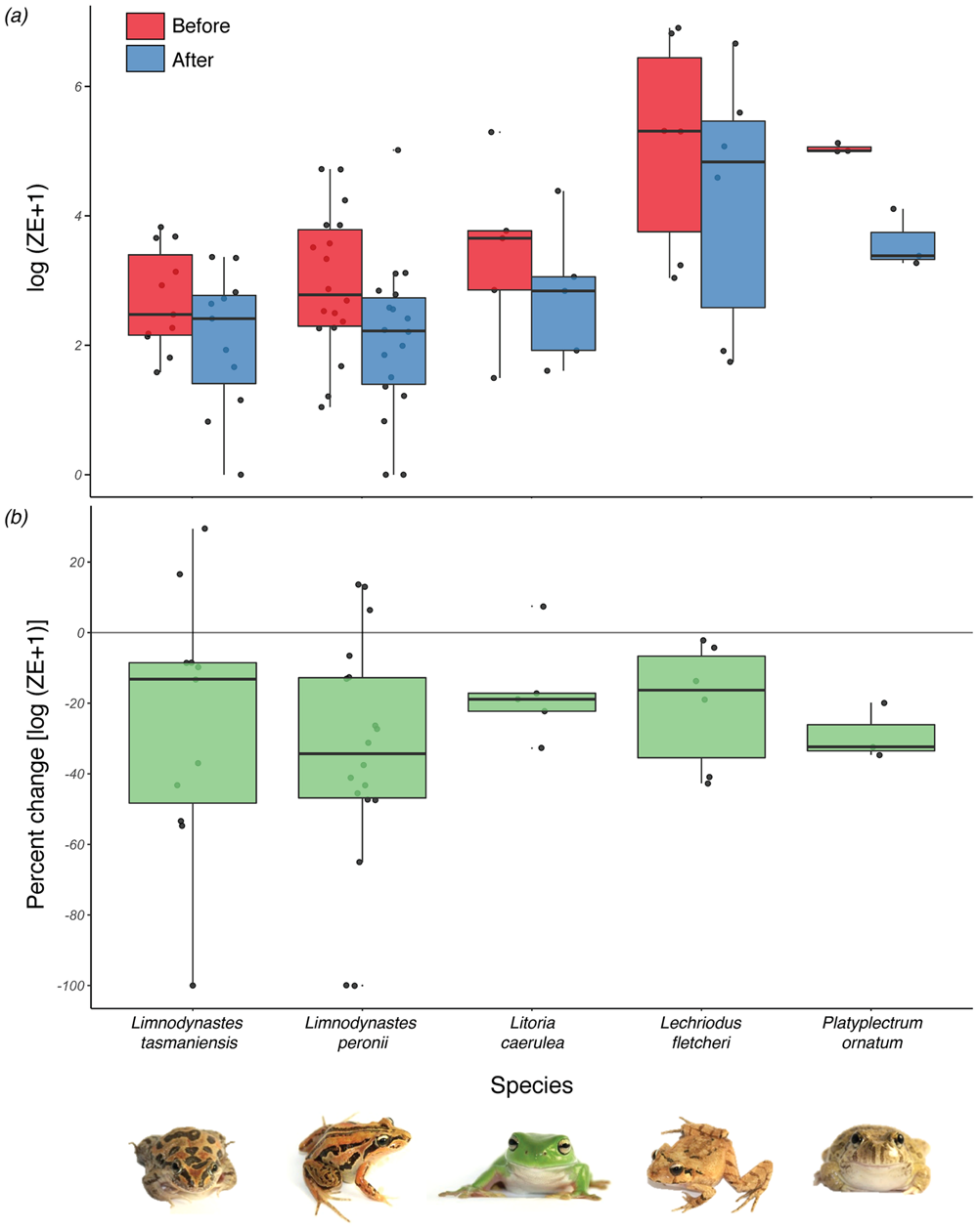
(**a**) Boxplots of infection load (log [zoospore equivalents (ZE) + 1]) before and after sloughing, and (**b**) percent change (log [ZE + 1]) in pathogen load from before to after sloughing in five Australian frog species infected with *Batrachochytrium dendrobatidis* (*Bd*). The centre line is the 50^th^ percentile, top and bottom of box represent 75^th^ and 50^th^ percentile, and whiskers extend to extreme data points (no more than 1.5 times the interquartile range). Photographs by M.E.B. Ohmer.

**Figure 3:**
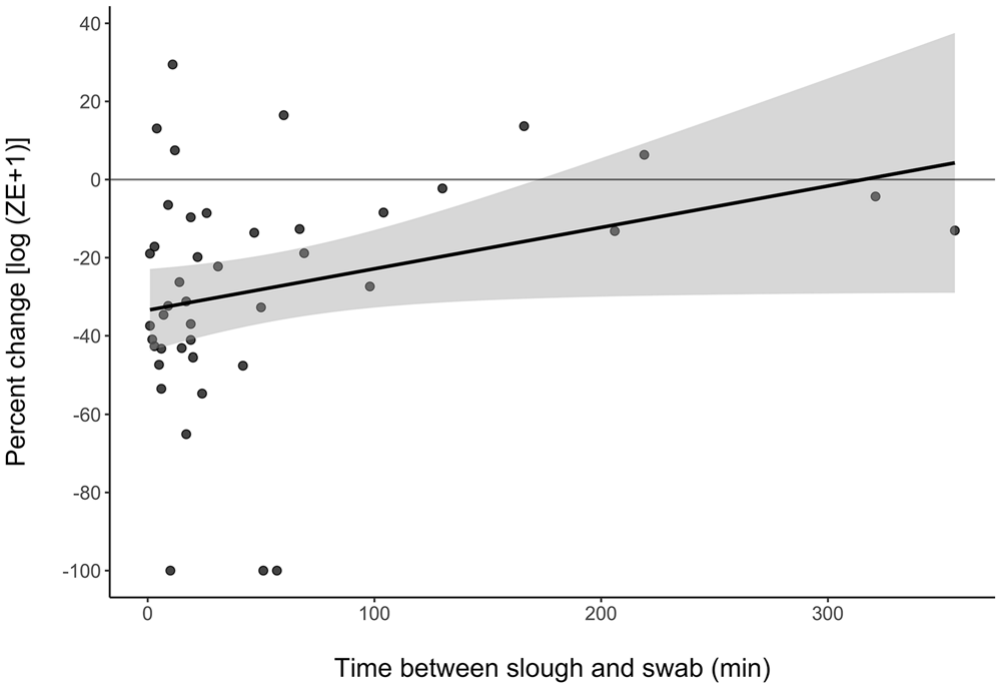
The association between the percent change in infection load (log [zoospore equivalents (ZE) + 1]) and the time (min) between a sloughing event and the ‘after’ swab, in frogs infected with the pathogen *Batrachochytrium dendrobatidis* (*Bd*). Shaded area indicates standard error.

To assess *Bd* load before and after sloughing, swabbing of individual frogs occurred between 10 and 386 min before (mean: 104.9 ± 102.8 s.d. min) and 1 and 356 min after (mean: 55.2 ± 82.4 s.d. min) each sloughing event. *Bd* load decreased in all species following sloughing (all species: F = 50.8, d.f. = 1, 43.1, *p* = 8.34 × 10^−9^, see Supplementary Table S5 for phylogenetic linear mixed model (PLMM) and individual species mixed model details, Fig. 2a). The percent change in *Bd* load (log[ZE + 1]) from before to after sloughing was not significantly different between species over time (F = 0.38, d.f. = 4, 2.9, *p* = 0.81, Supplementary Table S6, Fig. 2b), but was marginally positively associated with the *time between (min)* the sloughing event and the after swab (F = 4.02, d.f. = 22, *p* = 0.057; Fig. 3). This indicated that the sooner the frog was swabbed after sloughing, the greater the percent decrease in *Bd* load was observed. In *Lim. tasmaniensis* and *Lim. peronii*, species that experienced low mortality rates after *Bd* exposure, sloughing events sometimes resulted in a 100% reduction in *Bd* load (twice in *Lim. peronii* and once in *Lim. tasmaniensis*), and infection clearance occurred in those individuals.

### Change in sloughing rate with Bd infection

**Figure 4:**
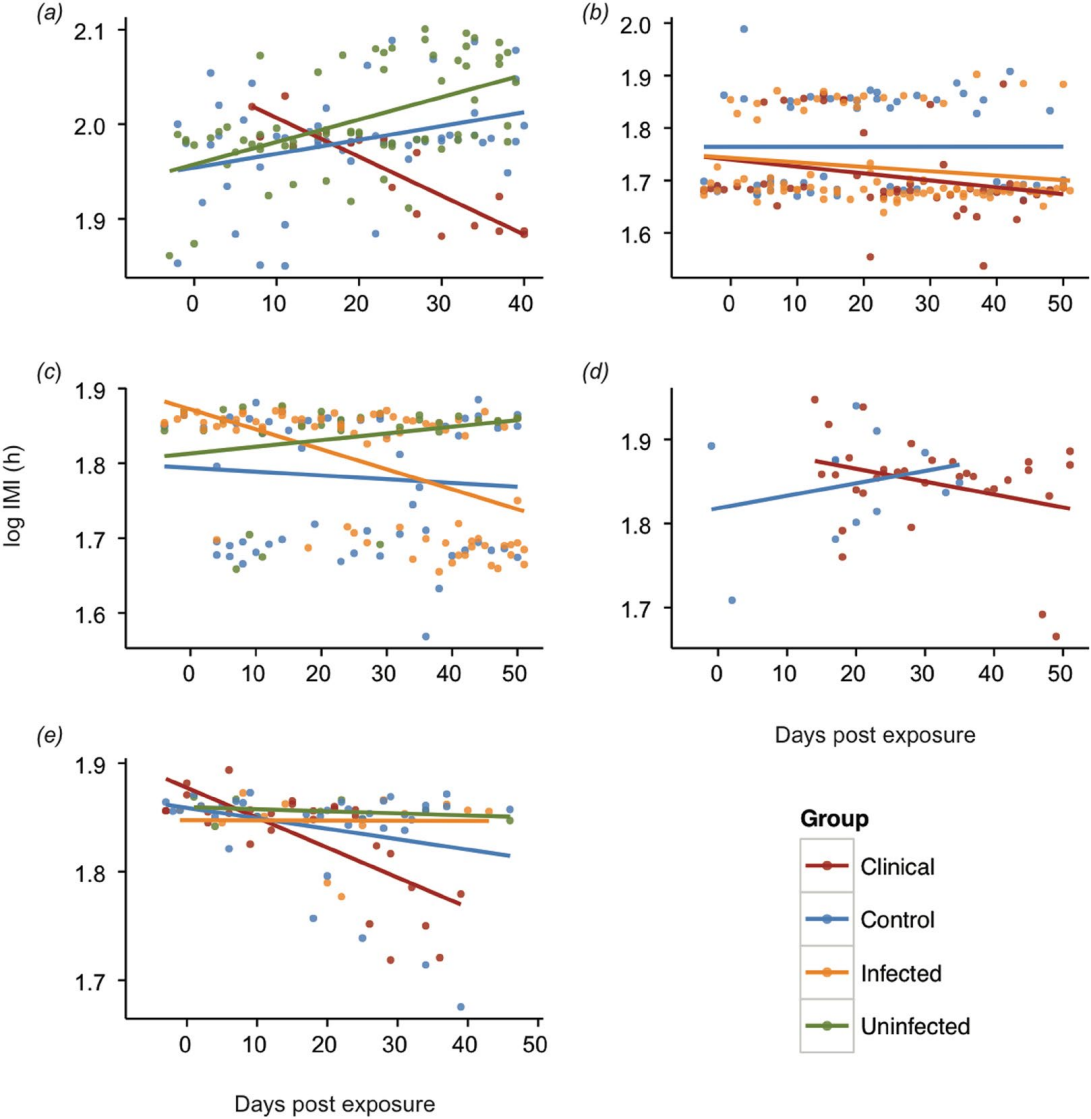
Log intermoult interval (IMI, h) for the first 40 days post exposure in *Litoria caerulea* (**a**), and the first 50 days post exposure in (**b**) *Limnodynastes peronii*, (**c**) *Lim. tasmaniensis*, (**d**) *Platyplectrum ornatum*, and (**e**) *Lechriodus fletcheri*. Colours indicate group based on disease outcome (control, clinical, infected, or uninfected). Data for *Lit. caerulea* is truncated before the third experimental exposure to *Batrachochytrium dendrobatidis* (*Bd*) because exposure groups changed. Longitudinal data was sparser for *P. ornatum* due to their burrowing behaviour.

**Figure 5:**
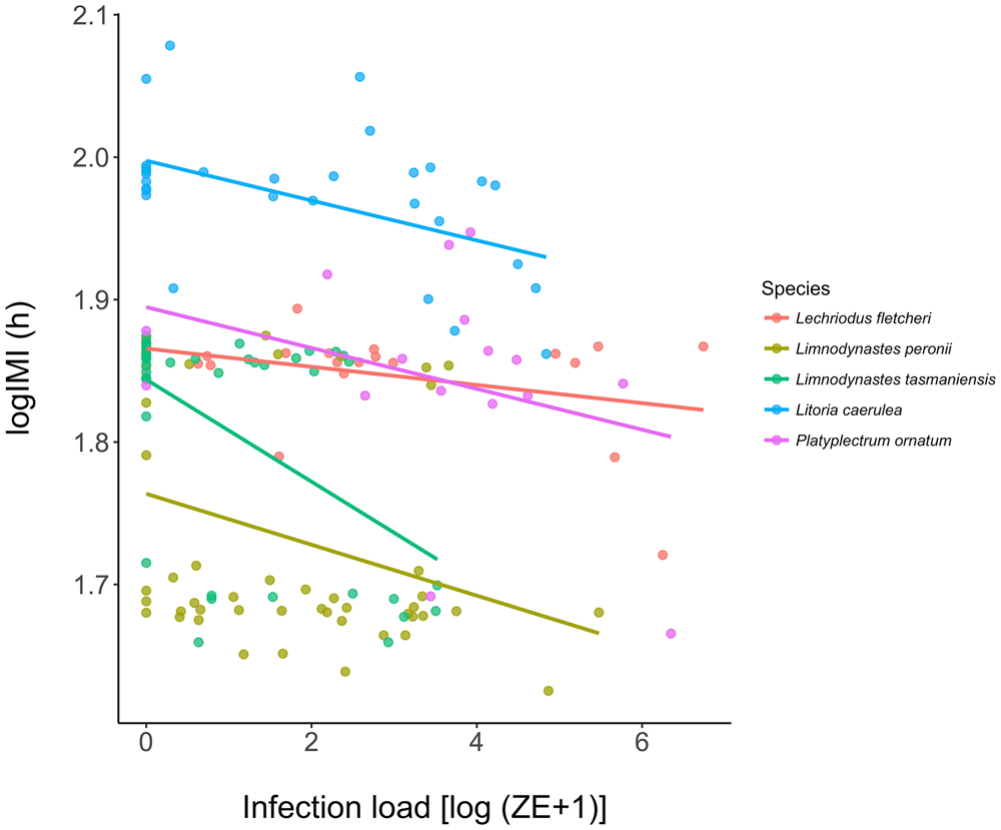
Change in log intermoult interval (IMI, h) with *Batrachochytrium dendrobatidis* (*Bd*) infection load (log [ZE + 1]) in infected individuals of five frog species.

On average, log IMI decreased in clinically infected *Lit. caeruela* (t = −7.2, d.f. = 121, *p* < 0.0001) and *Le. fletcheri* (t = −2.51, d.f. = 78, *p* = 0.014), and in non-clinically infected *Lim. tasmaniensis* (t = −3.72, d.f. = 160, *p* = 0.0003), indicating an increase in sloughing rate over time in these groups (Fig. 4, Supplementary Table S7). In *Lit. caerulea*, pairwise comparisons revealed that the decrease in log IMI over time in clinically infected animals was significantly different from both control (Χ^2^ = 54.2, d.f. = 1, *p* = 3.71 × 10^−13^) and uninfected animals (Χ^2^ = 73.9, d.f. = 1, *p* < 2.2 × 10^−16^). In *Le. fletcheri*, the decrease in log IMI over time in clinically infected animals was significantly different from control (Χ^2^ = 6.9, d.f. = 1, *p* = 0.042), uninfected animals (Χ^2^ = 6.9, d.f. = 1, *p* = 0.04), and infected animals (Χ^2^ = 10.8, d.f. = 1, *p* = 0.006). In *Lim. tasmaniensis*, the decrease in log IMI over time was significantly different between control and infected animals (Χ^2^ = 14.3, d.f. = 1, *p* = 0.0003), and infected and uninfected animals (Χ^2^ = 19.6, d.f. = 1, *p* = 2.81 × 10^−5^), but not control and uninfected animals (Χ^2^ = 1.52, d.f. = 1, *p* = 0.22). There was no difference in sloughing rate amongst groups in *Lim. peronii* and *P. ornatum* (Fig. 4, Supplementary Table S7). Furthermore, when species and phylogenetic structure are included as random effects, infected frogs demonstrated an increase in sloughing rate with infection load, as demonstrated in *Lit. caerulea* previously (F = 20.79, d.f. = 1, 145.6, *p* = 1.08 × 10^−5^; Fig. 5, Supplementary Table S8A, ref. 18). Interestingly, sloughing rate in infected *Lim. tasmaniensis* increased at lower *Bd* infection loads than the other two species that increased their sloughing rates in the clinical group (*Li. caerulea* and *Le. fletcheri*) (Fig. 5, Supplementary Table S8B), as demonstrated by a significantly steeper slope in pairwise comparisons of the relationship between *Bd* load and IMI, and this species demonstrated the lowest susceptibility to chytridiomycosis (0% mortality).

## Discussion

Amphibians regularly shed their skin, and the importance of this frequent, sometimes daily, process has heretofore been overlooked. By comparing the sloughing rates and infection loads across five frog species with different susceptibilities to *Bd* infection, we were able to demonstrate that skin sloughing does indeed reduce *Bd* load on the ventral skin surface, and can even result in infection clearance. However, this decrease in *Bd* load on the skin surface is only temporary in susceptible species, and many individuals still developed clinical disease. Infection intensity, mortality, and skin sloughing rates varied across host species, as did the timing of increase in those sloughing rates. It is likely that sloughing is a double-edged sword: sloughing reduces *Bd* load, and can aid in clearance of infection in some individuals, yet, skin sloughing is a physiologically vulnerable time for amphibians^19^, and an increase in sloughing rate at high infection loads may be more detrimental than beneficial.

Utilizing a refined methodology (live video feeds) to swab frogs as soon as possible after sloughing, and avoiding the confounds of the swabbing action itself by swabbing only half the frog, we found that the mechanical action of removing the *stratum corneum* does indeed reduce *Bd* loads on the skin surface across all species. In *Lim. peronii* and *Lim. tasmaniensis*, species that experienced low mortality rates after *Bd* exposure, sloughing sometimes resulted in 100% reduction in *Bd* load, and eventual clearance of infection. This indicates that sloughing can act as a defence to limit *Bd* infection load. However, in species that suffered high mortality rates, such as *P. ornatum* and *Le. fletcheri*, sloughing did not permanently reduce *Bd* loads, and infection intensity continued to increase. These host-dependent differences may be a reflection of the variation in *Bd* population growth patterns on an amphibian’s skin^21^. If the growth of *Bd* remains epibiotic, as seen in skin explants of the tolerant species *Xenopus laevis* ^21^, then sloughing may be more effective at removing encysted zoospores and resulting zoosporangia. This may be the case in *Lim. tasmaniensis*, in which 71% of individuals became infected, but over half of all individuals cleared infection by the end of the experimental period, and no clinical signs of infection were observed (e.g. inappetence, weight loss, or abnormal skin shedding). In the context of this experimental infection, *Lim. tasmaniensis* may be considered a species resistant to *Bd* infection, given that it can reduce pathogen colonization and/or invasion until clearance^27^.

Organisms can defend themselves from a pathogen via two mechanisms, which are not mutually exclusive: resistance and tolerance^27, 28^. While resistance mechanisms prevent infection or limit pathogen growth, tolerance mechanisms limit fitness effects of a given pathogen burden. Strictly speaking, because skin sloughing can limit pathogen growth, it would be defined as a resistance mechanism in competent host species that are able to limit the invasion of *Bd* in the epidermis via other immune defences, such as antimicrobial skin peptides or antimicrobial metabolites^25, 29, 30^. However, in susceptible hosts, it would appear that sloughing is not enough to remove *Bd* infection on its own, and this may be due to the invasive nature of *Bd* growth in these species^21^. Sloughing may also play a role in resistance to *Bd* colonization. If sloughing occurs very soon after *Bd* exposure, it may rid the host of the pathogen completely before invasive *Bd* growth occurs. Yet, Van Rooij *et al*.^21^ found that invasive germ tubes were seen as soon as two hours post exposure in *Lit. caerulea*, and by 16 hours post exposure, chytrid thalli were growing intracellularly^21^. In a previous experimental exposure, however, no association was found between *Bd* exposure and the timing of first slough and infection outcome in *Lit. caerulea* ^18^.

Sloughing could also be seen as playing a role in allowing host tolerance to *Bd* infection, by regulating *Bd* infection spread and thereby limiting the health effects of infection^27^. In some host species tolerant of high infection loads, such as *Pseudacris regilla* in North America, it has been hypothesized that tolerance arises from localized infections isolated to certain patches on the skin, despite intracellular growth^31^. Mechanisms that restrict *Bd* from spreading to other skin areas are not known, but sloughing could play a role in preventing spread by removing zoospores released onto the skin from nearby infected areas.

It was previously found that frogs infected with *Bd* have increased sloughing rates, but only at high infection loads^18^. We discovered a similar trend in this study, with infected individuals of all species increasing their sloughing rates, except in *Lim. peronii* and *P. ornatum*. The lack of sloughing rate plasticity in *Lim. peronii* may be due to the already fast baseline sloughing rate, or perhaps a lower number of replacement cell layers in the epidermis precludes sloughing plasticity^24^. In *P. ornatum*, however, the lack of a significant increase in sloughing rate in infected frogs may be attributable to the difficulty in observing these burrowing animals at all times, thereby reducing the ability to detect a longitudinal trend. Most interestingly, the least susceptible species, *Lim. tasmaniensis*, increased sloughing rates at lower infection loads, and over half of individuals eventually cleared infection. This indicates that sloughing may serve as an effective defensive response and not only as a baseline defence in this species, because sloughing rate increased before a high level of cutaneous *Bd* population growth on the skin was reached. Despite evidence that *Bd* evades the immune response and/or results in immunosuppression in some species^32, 33^, an increased sloughing rate in *Lim. tasmaniensis* may indicate a defensive response, although the mechanisms underlying this response are unknown. Future work should investigate increased sloughing as a principal mechanism of defence in other amphibian species known to be resistant to *Bd* infection.

Resistance to infection can be costly, as an increased immune response can result in not only damage to the pathogen, but also the host (termed immunopathology; reviewed in refs 34 and 35). While we have shown that skin sloughing can reduce *Bd* loads and even clear infection in some hosts, sloughing itself leaves amphibians physiologically vulnerable. It has been shown that skin permeability to water and sodium increases during the sloughing process, temporarily disrupting physiological homeostasis^19, 36^. In frogs with severe chytridiomycosis, water and electrolyte imbalances are signs of clinical disease^20, 37^. Furthermore, infected frogs experience high cutaneous water loss rates during sloughing (C. Russo, unpublished data), and may be slow to rehydrate^38^, corroborating findings of dehydration in severely infected frogs^37, 39^. Finally, sloughing also reduces bacterial populations on the skin^13^, and may actually disrupt the re-colonization of beneficial symbiotic bacteria that have been shown to produce antimicrobial metabolites effective against *Bd* ^40, 41^. Thus, an increase in sloughing rate at advanced infection stages may cause more harm than good, exacerbating imbalances in fluid and electrolyte levels^18^. In *Lim. tasmaniensis*, however, sloughing rate increased at lower infection loads, perhaps limiting the negative effects of increased sloughing while increasing the rate of pathogen clearance. Future research avenues should investigate whether sloughing coincides with the renewal of additional innate immune defence mechanisms, such as host peptide defences, which have been shown to play a role in defence against *Bd* infection^25^.

The five Australian frog species exposed to *Bd* in this experiment demonstrated wide variation in susceptibility to *Bd* infection and chytridiomycosis. However, this variation may not be suggestive of *Bd* infection in wild populations of these species. In this study, frogs were kept in high-humidity conditions that were not indicative of typical conditions experienced by all of these species in the wild. In particular, *P. ornatum* is often found in dry or semi-arid conditions far from a permanent water source^42^, thus high inherent susceptibility to disease under optimal conditions for the pathogen (as in this study) may not relate to population-level effects in an environmental context. This has been demonstrated in *Lit. caerulea*, which is typically very susceptible to *Bd* infection and chytridiomycosis in laboratory settings^18, 20^, but has not undergone significant declines in the wild^6, 43, 44^. Unexpectedly, adult *Lit. caerulea* collected for use in the current study demonstrated low infection prevalence and low mortality rates after exposure to *Bd*, in comparison to our previous study of this species^18^. This may be due to the fact that in the current study frogs were collected from a population only 15 km north of previous records of *Lit. caerulea* with *Bd* infection^45^, despite all animals being *Bd* negative upon collection. While there is currently no direct evidence of individuals from populations with long histories of *Bd* infection evolving resistance^46^, repeated exposures to *Bd* and subsequent heat treatment has conferred increased disease resistance in at least one species in the laboratory^47^. In addition, *Lit. caerulea* in the current study had been in captivity for a shorter period of time than in our previous study, which may also have contributed to the differences in *Bd* susceptibility (immunocompetence) observed. For example, immune defences such as the cutaneous bacterial community have been shown to be significantly different in captive animals, and this may influence disease susceptibility^48^. Finally, when comparing *Lit. caerulea* to the other three species, it is important to keep in mind that they were collected as adults, while the other three were raised from spawn in captivity. Although there is little data on how rearing history influences subsequent *Bd* susceptibility in amphibians, it cannot be ignored as a potential factor contributing to the observed susceptibility differences across species. Therefore, this caveat should be kept in mind when comparing susceptibility across species.

In order to best utilize models of host extinction risk following *Bd* exposure, a better understanding of the host-pathogen relationship for model parameterization is required^26^. Demonstrating that sloughing can reduce *Bd* loads on both susceptible and resistant hosts has implications for understanding the epidemiology of this pathogen in wild populations. In establishing that sloughing can regulate *Bd* population growth, this cyclic process can be built into patterns of *Bd* growth on individual hosts, and the effects can be modelled at a population-level. In addition, these findings have implications for interpreting swab results collected from individuals at a single time point. In demonstrating that skin sloughing can indeed reduce *Bd* load on the epidermis in multiple frog species, sometimes up to 100%, we indicate the potential for false negatives, or an underestimation of actual infection load, if swabbing occurs shortly after sloughing. Many studies have reported frogs gaining and losing infection over short time scales^31, 49, 50^, and this may be in part due to amphibian skin sloughing.

Differences in susceptibility to *Bd*, a generalist pathogen implicated in the decline or extinction of over 200 amphibian species worldwide^7^, may be linked to inherent differences in the amphibian epidermis. This study demonstrates that amphibian skin sloughing, which varies in rate across species and increases with temperature^13, 14^ and disease progression^18^, can also regulate *Bd* population growth. The efficacy of the regulation of *Bd* population growth is host-dependent, however, and indicates a key difference in the role of sloughing as a skin defence mechanism in susceptible versus tolerant or resistant hosts. This work has significant conservation implications, as it may improve our predictions of host-specific responses to *Bd* in wild populations, allowing for better conservation planning.

## Materials and Methods

### Ethics statement

All methods involving animals were approved by and carried out in accordance with the guidelines and regulations of permit SBS/452/12/URG issued by the University of Queensland Animal Welfare Committee and permit WISP12218412 issued by the Queensland Environmental Protection Agency.

### Study species

In order to examine a range of susceptibilities to *Bd* infection and subsequent disease, we compared sloughing rates and infection loads of five fairly common species of frog from southeastern Queensland: spotted and striped marsh frogs (*Limnodynastes tasmaniensis* and *Lim. peronii*), ornate burrowing frogs (*Platyplectrum ornatum)*, black-soled frogs (*Lechriodus fletcheri*), and green tree frogs (*Litoria caerulea*). *Bd* infections have been previously recorded in wild populations of all of these species^43, 44^, but there is no definitive evidence of disease-related declines in these species. Previous exposure experiments indicate that *Lit. caerulea* is susceptible to chytridiomycosis in the laboratory^18, 20^, while *Lim. tasmaniensis* and *Lim. peronii* demonstrate fairly low susceptibility^25, 51^. There are no published studies in which *Le. fletcheri* has been exposed to *Bd* in a laboratory setting.

### Animal collection and maintenance

*Lim. tasmaniensis, Lim. peronii*, *P. ornatum*, and *Le. fletcheri* were collected as spawn in southeastern Queensland, Australia and reared in the laboratory (for additional details, see Supplementary Materials). At the time of experimentation, all frogs had reached adult size and were about two years of age, with the exception of *Le. fletcheri*, which had only reached subadult size, and were about 1.5 years old (Supplementary Table S1a). Adult *Lit. caerulea* were collected from wet roadsides in non-protected areas near Fernvale, Queensland. Frogs of a single species originated from one population, eliminating the need to account for population or site differences within a species. In the laboratory, frogs were kept on a cycling temperature regime (15–23 °C) with a 12 h photoperiod (see ref. 18 for detailed temperature cycle). Frogs were housed individually in ventilated clear plastic boxes (*Lit. caerulea:* 262 × 237 × 120 mm, *Le. fletcheri*: 172.5 × 120 × 75 mm, all others: 235 × 170 × 120 mm), with a substrate of paper towels saturated with aged tap water and a plastic cup or PVC pipe for shelter. Enclosures were cleaned and frogs were fed vitamin and calcium-dusted crickets (5 large or medium, depending on frog size), weekly. All frogs tested negative for *Bd* infection prior to the start of the experiment.

### Exposure to Bd and infection monitoring

Frogs were exposed to *Bd* strain EPS4, which was isolated by E.P. Symonds (School of Veterinary Sciences, The University of Queensland) in March 2012 from a *Mixophyes fleayi* tadpole (Gap Creek, Main Range National Park, Queensland, Australia). For additional details of animal exposure to *Bd*, see supplementary materials and Table S1.

At two days and seven days following each *Bd* exposure, all animals were swabbed to assess infection status. Swabbing protocol followed Ohmer *et al*.^18^, with the exception of swabs taken before and after sloughing, which followed a specific protocol to avoid any potential artefacts from the swabbing itself (see *Monitoring infection load before and after sloughing*). Subsequently, animals were swabbed approximately every two weeks, and swabs were analysed with quantitative PCR (qPCR) following Boyle *et al*.^52^ and Hyatt *et al*.^53^ (see Supplementary Materials for additional details).

### Sloughing monitoring

Frogs were recorded continuously with twelve 600TVL Weatherproof infrared security cameras (model EN-CI20B-65H, Eonboom Electronics Limited) at a frame rate of 1.52 frames per second (FPS). Video was recorded on a 16 Channel H.264 Digital Video Recorder (DVR), model MDR688ZB (AU)-E. 600TVL). Sloughing behaviour is unique and easy to recognize on recorded video when played back at 16x normal speed (see Ohmer *et al*.^18^ for example recordings).

### Monitoring infection load before and after sloughing

Frog sloughing rates were analysed from recorded video, in order to predict their sloughing rhythm. Individual frogs often slough on an anticipated cycle and at a consistent time of day in the laboratory, allowing for sloughing events to be predicted with some accuracy. Frogs were swabbed as close as possible to this predicted sloughing time, and then swabbed again as soon as possible after sloughing occurred. To achieve this, surveillance cameras were networked, allowing the remote viewing of frog behaviour as it occurred (a methodological update from^18^). To avoid any artefact from swabbing itself, only one side of the ventral surface of each frog (right or left, divided along the sagittal plane) was swabbed before sloughing (randomly chosen), and the opposite side was swabbed after sloughing. When swabbing one side, swabs were run up and down the left or right ventral surface including the drink patch, the thigh, the side of the torso, one fore foot and one hind foot, three times each.

### Phylogenetic relationships

A phylogenetic tree of relationships between species in this study was obtained from the Open Tree of Life^54^, accessed via the R package ‘rotl’^55^, and Grafen’s arbitrary branch lengths were used for tree creation (ref. 56; Supplementary Fig. S1).

### Statistical Analyses

All statistical analyses were performed in the program R^57^. Phylogenetic linear mixed models (PLMMs), implemented in ASReml-R, were utilized to account for multiple measurements on the same individuals over time, and phylogenetic non-independence between species (function ‘asreml’, package ‘asreml’, ref. 58). For all PLMMs, a Wald type F-test was used to test for the significance of fixed effects^59^, and the significance of random effects were determined using likelihood ratio tests^60, 61^. Approximate standard errors for the estimate of phylogenetic heritability were calculated using the R pin function^62^. Phylogenetic heritability is equivalent to the more widely-used λ^63^, and was used as an estimate of phylogenetic signal. Phylogenetic heritability was calculated as the proportion of the variance in the trait, conditioned on the fixed effects^64^, which is explained by the relationship among taxa as given by the phylogeny^65^. PLMMs were reduced using likelihood ratio tests^60, 61^.

In order to examine the change in infection load (log[ZE + 1]) over time in *Bd* exposed frogs, a PLMM was fitted with the interaction between *Species* and *Days post exposure*, and *Days post exposure* ^2^, as the fixed effects, and *Frog ID* nested within *Species* and a phylogenetic variance-covariance matrix constructed from the phylogeny as random effects. Survival curves were compared between species with a log-rank test (function ‘survdiff’, package ‘survival’). The natural log of slough duration (min) was also compared across species, with *Days post exposure* and *Group* as fixed effects, and the same random effects as the previous model.

To test for a change in *Bd* load (log [zoospore equivalents (ZE) + 1]) after sloughing, a PLMM was performed, including individual *Frog ID* nested within *Species, Days post exposure*, and a phylogenetic variance-covariance matrix constructed from the phylogenetic tree as random effects, and *Before or After* (sloughing) as the fixed effect. Furthermore, individual mixed-effects models were run for each species to examine within species effects of sloughing on Bd load. To examine the role of swabbing timing after sloughing in the observed percent change in *Bd* load, we used a PLMM to examine the percent change in *Bd* load (log[ZE + 1]) from before to after sloughing, with the same random effects as the previous model, and the interaction between *Days post exposure* and *Species* as the fixed effects.

To explore differences in the change in log IMI for each species during the experiment, separate linear mixed effects models with *Days post exposure* (‘day’ indicating the first slough date of consecutive sloughs between which an IMI was calculated), and *Group* (Control, Infected, Uninfected, or Clinical) as fixed effects, and *Frog ID* as a random effect to account for multiple measurements on the same individuals over time, were performed (function ‘lme’, library ‘nlme’, using Maximum Likelihood^66, 67^). *Days post exposure* was used as a proxy for time progression in the experiment, due to missing values for a few sloughing events in individual frogs (particularly those of the burrowing species, i.e. *P. ornatum*). IMI is the time in hours between sloughing events, with shorter IMIs indicating a faster sloughing rate. Pairwise comparisons of the relationship between *IMI* and *Days post exposure* were then performed between groups (function ‘testInteractions’, library ‘phia’). With the same random effects as previous PLMMs, the change in IMI with *Bd* load (log [ZE + 1]) was also compared across all species. Finally, a mixed effects model was used to compare the species that demonstrated significant increases in their sloughing rate in the clinical or infected groups (random effect: *Frog ID*), and post-hoc comparisons of the interaction between *Species* and *Bd* load were performed (function ‘testInteractions’, library ‘phia’).

### Data availability

The datasets generated and analyzed during the current study are available in the Dryad digital repository doi:10.5061/dryad.j464h.

## Electronic supplementary material

Supplementary materials
