# Supplementary material for: Skin sloughing in susceptible and resistant amphibians regulates infection with a fungal pathogen
Source: Sci Rep. 2017 Jun 14;7:3529. doi: 10.1038/s41598-017-03605-z (PMC5471217; doi:10.1038/s41598-017-03605-z)
Supplement: Supplementary file 1 — Supplementary materials [file 41598_2017_3605_MOESM1_ESM.pdf]

Electronic supplementary material

Skin sloughing in susceptible and resistant amphibians regulates infection with a fungal pathogen

Authors: Michel E. B. Ohmer, Rebecca L. Cramp, Catherine J. M. Russo, Craig R. White, Craig E. Franklin

Methods- Additional details

Animal collection

Egg masses of *Lim. tasmaniensis* and *P. ornatum* were collected from flooded roadsides near Dalby, Queensland while egg masses of *Lim. peronii* were collected from ephemeral pools in St. Lucia, Queensland. Egg masses of *Le. fletcheri* were collected from pools on private land on the Lamington plateau, Canungra, Queensland, with permission.

Bd exposure and infection monitoring

Bd culture was maintained in flasks at 4˚C until four days before exposure, when it was re-cultured onto 1% agar, 0.25% tryptone, 0.25% tryptone-soy plates for 5-7 days at 21˚C. Once zoospore production peaked, plates were flooded with distilled water for 30 min with periodic gentle agitation. The resulting suspension of zoospores was collected, and zoospore concentration was calculated using a haemocytometer ([55]).

All frogs were experimentally exposed to *Bd* at least twice to ensure infection, on 8 April 2015 at a dose rate of ~250,000 zoospores, and two weeks later on 22 April 2015 at a dose rate of ~500,000 zoospores (Table 1a). Green tree frogs were exposed a third time, on 20 May 2015, with a different strain of *Bd* (strain *Waste point-Lverreauxii-2013-LB,RW,2*, isolated by Lee Berger at James Cook University, dose: 500,000 zoospores), in order to attain a greater number of infected individuals (Table 1b). Exposure protocol followed that of Ohmer et al [23], with

frogs exposed for five hours in 300 mL plastic containers containing 40 mL of aged tap water (see Table 1 for exposure groups). Control frogs were treated in the exact same manner as exposed frogs, but were exposed to aged tap water containing no zoospores.

All frogs were monitored daily for clinical signs of disease. If a frog began to demonstrate severe clinical signs, including lethargy, inappetence, abnormal posture (particularly with legs splayed out and head bent over toward substrate), and poor righting reflex, a final swab was taken to determine infection load, and the animal was humanely euthanized in 0.3% neutral buffered MS-222. Clinically infected *Lit. caerulea* were euthanized with an intracoelomic injection of 60 mg kg^{-1} body mass thiopentone sodium (Ilium Thiopentone, Troy Laboratories, NSW, Australia), to allow for additional studies of the skin post-mortem.

Swabs were analyzed with quantitative PCR (qPCR) following Boyle et al [55] and Hyatt et al [56]. Briefly, swabs were extracted in 50 µL Prepman Ultra (Applied Biosystems, Foster City, CA, USA) and analyzed in triplicate with qPCR on a Mini Opticon real-time PCR detection system (MJ Mini Cycler, Bio-Rad Laboratories, Inc.). Infection load was determined by multiplying by 100 to account for dilution and expressed as zoospore equivalents (ZE). A modified 15-µL reaction volume was used [23, 57].

Table S1. (a) Exposure groups and descriptive statistics for five Australian frog species exposed to *Batrachochytrium dendrobatidis* (*Bd*). All species were exposed to *Bd* strain EPS4 twice. (b) Details of third experimental exposure to *Batrachochytrium dendrobatidis* (*Bd*) for *Litoria caerulea* only. Exposure 3 utilized strain *Waste point-Lverreauxii-2013-LB, RW, 2*, and exposure and control groups were re-organized to attain a greater number of infected individuals. *L. caerulea* in the 'new controls' group were previously exposed animals that never became infected, those in the 'new exposed' were previously controls, and two animals ('not re-exposed') were demonstrating signs of chytridiomycosis and not included in the third exposure

(a)

**Species**	**Control (n)**	**Exposed (n)**	**SVL (mm) mean ± s.d.**	**Life stage**
Lechriodus fletcheri	3	4	26.8 ± 2.2	Subadult
Limnodynastes peronii	3	6	38.6 ± 1.4	Adult
Limnodynastes tasmaniensis	4	7	35.6 ± 3.1	Adult
Litoria caerulea	6	11	83.2 ± 5.7	Adult
Platyplectrum ornatum	4	6	36.5 ± 3.5	Adult

(b)

**Group (n)**	**Exposure 3 (*L. caerulea* only)**
Control	3
Exposed	5
New control	4
New exposed	3
Not re-exposed	2

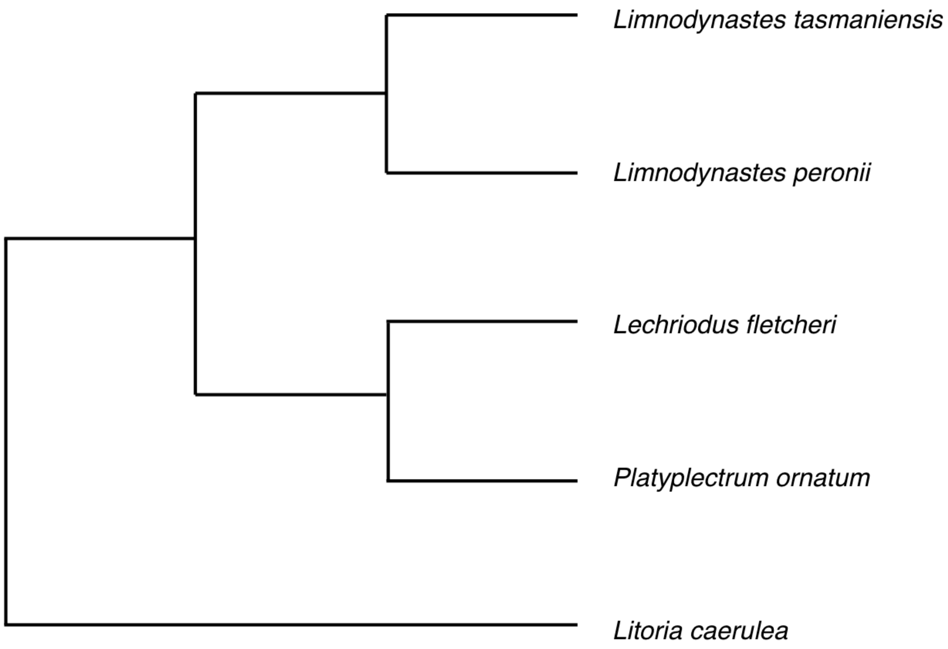

Fig. S1. Phylogenetic relationships between the species included in this study, displayed with Grafen’s arbitrary branch lengths.

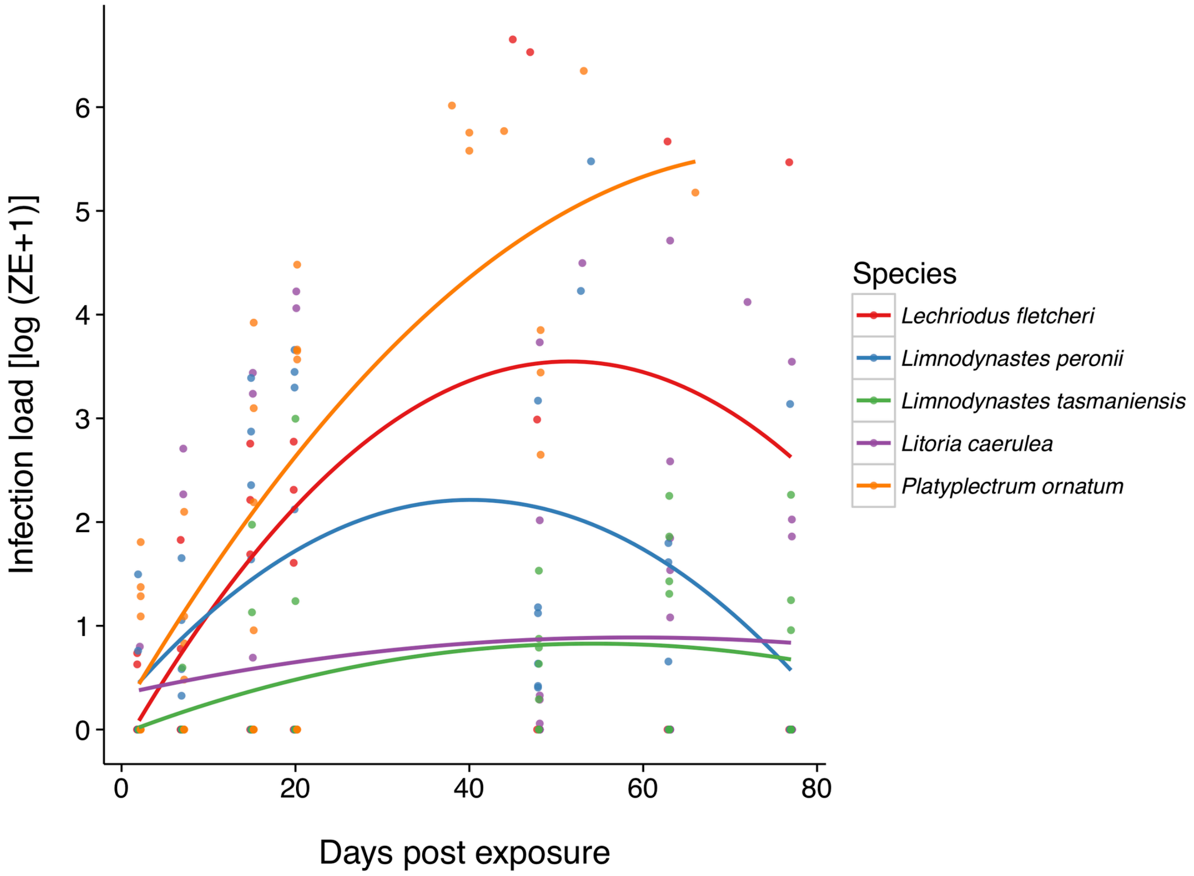

Fig. S2. Change in infection load (log [zoospore equivalents (ZE)+1]) after exposure to *Batrachochytrium dendrobatidis* (*Bd*) in five frog species found in Southeast Queensland, Australia. Curves are quadratic polynomial smoothing functions fit by species**.**

S2 Table. Mean prevalence over the experimental period (first 40 days only for *Litoria caerulea*, before exposure groups changed), mortality rates, and the percentage of animals that cleared infection after becoming infected for five frog species exposed to *Batrachochytrium dendrobatidis*. s.d. = standard error

Species	N exposed	Mean prevalence	s.e.	Mortality rate	Percent cleared
Lechriodus fletcheri	4	57.1	6.1	50%	0%
Limnodynastes peronii	6	61.9	10.4	33%	50%
Limnodynastes tasmaniensis	7	34.7	9.3	0%	60%
Litoria caerulea	11	21.8	3.1	18%	25%
Platyplectrum ornatum	6	73.3	6.1	100%	0%

S3 Table. Statistical results from a phylogenetic linear mixed model (implemented in ASReml-R) examining the change in infection load (log (zoospore equivalents [ZE]+1)) during the experimental period in infected frogs only of five species of Australian frog exposed to *Batrachochytrium dendrobatidis* (*Bd*). Fixed effects were the interaction between *Species* and *Days post exposure,* and *Days post exposure*^{2}, and random effects were *Frog ID* nested in *Species,* and a variance-covariance matrix created from the phylogeny, to take into account correlated error from repeated measures on the same individual, and phylogenetic non-independence. s.e. = standard error, d.f. = degrees of freedom, bold p-values are significant, λ = phylogenetic signal

**Fixed effects**	**Estimate**	**s.e.**	**F**	**d.f.**	***p***
Intercept	0.070	0.25	51.55	1, 126.6	0.78
Species: Days post exposure (*Lechriodus fletcheri*)	NA	NA	17.85	4, 201.9	**1.5×10^{-12}**
Species: Days post exposure (*Limnodynastes peronii*)	-0.19	0.052			
Species: Days post exposure (*Limnodynastes tasmaniensis*)	-0.21	0.050			
Species: Days post exposure (*Litoria caerulea*)	-0.21	0.047			
Species: Days post exposure (*Platyplectrum ornatum*)	0.21	0.068			
Days post exposure	0.44	0.078	30.31	1,187.4	**6.6×10^{-9}**
Days post exposure^2	-0.012	0.0041	25.0	1,187.9	**4.9×10^{-3}**
**Random effects**	**Variance**	**s.e.**	**χ^2**	**d.f.**	***p***
Phylogeny	1.01×10^{-7}	1.24×10^{-8}	-7.6×10^{-7}	1	1
Frog ID nested in Species	0.71	0.25	NA	NA	NA
**λ = 5.92×10^{-8}, se = 7.45×10^{-9}**					

S4 Table. Statistical results from a phylogenetic linear mixed model (implemented in ASReml-R) examining the natural log of sloughing duration (min) in five species of Australian frog exposed to *Batrachochytrium dendrobatidis* (*Bd*). Fixed effects were *days post exposure*, and *group* (control, clinical, infected and uninfected) and random effects were *Frog ID* nested in *Species,* and a variance-covariance matrix created from the phylogeny, to take into account correlated error from repeated measures on the same individual, and phylogenetic non-independence. s.e. = standard error, d.f. = degrees of freedom, bold p-values are significant, λ = phylogenetic signal

**Fixed effects**	**Estimate**	**s.e.**	**F**	**d.f.**	***p***
Intercept	2.15	0.056	4632.0	1, 2.8	**3.9×10^{-5}**
Days post exposure	0.0015	0.0012	1.53	1, 619	0.22
Group (Control)	NA	NA	1.34	3, 41	0.28
Group (Clinical)	0.068	0.070			
Group (Infected)	0.080	0.066			
Group (Uninfected)	-0.062	0.056			
**Random effects**	**Variance**	**s.e.**	**χ^2**	**d.f.**	***p***
Phylogeny	1.01×10^{-7}	1.12×10^{-9}	-7.36×10^{-7}	1	1
Frog ID nested in Species	7.21×10^{-2}	6.48×10^{-3}	11.82	1	**0.00059**
**$\lambda = 9.3 \times 10^{-8}$, s.e. = 3.2×10^{-9}**					

S5 Table. A) Statistical results from a phylogenetic linear mixed model (implemented in ASReml-R) examining the change in infection load (log (zoospore equivalents [ZE]+1)) after sloughing in five species of Australian frog exposed to *Batrachochytrium dendrobatidis* (*Bd*). Fixed effects were *before or after sloughing*, and random effects were *Frog ID* nested in *Species, days post exposure*, and a variance-covariance matrix created from the phylogeny, to take into account correlated error from repeated measures on the same individual, and phylogenetic non-independence. B) Individual mixed-effects models for each species, with the random effects *Frog ID* and *Days post exposure*, and the fixed effect *before or after sloughing.* s.e. = standard error, d.f. = degrees of freedom, bold p-values are significant, λ = phylogenetic signal

A) All species (phylogenetic model)

**Fixed effects**	**Estimate**	**s.e.**	**F**	**d.f.**	***p***
Intercept	2.96	0.68	24.76	1, 3.5	**1.01×10^{-2}**
Before or After	0.82	0.11	50.81	1, 43.1	**8.34×10^{-9}**
**Random effects**	**Variance**	**s.e.**	**χ^2**	**d.f.**	***p***
Phylogeny	3.71	0.96	2.26	1	0.13
Frog ID nested in Species	1.61	0.45	9.77	1	**0.0018**
Days post exposure	3.48	0.98	37.08	1	**1.13×10^{-9}**
**λ = 0.38, s.e. = 0.23**					

B) Individual species (mixed models)

Litoria caerulea

**Fixed effect**	**estimate**	**s.e.**	**t-value**	**d.f.**	***p***	**CI**	
						**2.5%**	**97.5%**
Intercept	3.46	0.83	4.14	4	**0.014**	1.38	5.53
Before or After	-0.65	0.19	-3.34	4	**0.029**	-1.14	-0.17
**Random effects:**	**s.d.**				**Residual**		
Frog ID	1.18						
Days post exposure/Frog ID	0.54				0.28		

Limnodynastes tasmaniensis

**Fixed effect**	**estimate**	**s.e.**	**t-value**	**d.f.**	***p***	**CI**	
						**2.5%**	**97.5%**
Intercept	2.70	0.29	9.46	10	**0.014**	2.09	3.30
Before or After	-0.62	0.23	-2.74	10	**0.021**	-1.10	-0.14
**Random effects:**	**s.d.**				**Residual**		
Frog ID	3.9×10^{-5}						
Days post exposure/Frog ID	0.75				0.50		

Limnodynastes peronii

**Fixed effect**	**estimate**	**s.e.**	**t-value**	**d.f.**	***p***	**CI**	
						**2.5%**	**97.5%**
Intercept	3.10	0.33	9.26	17	**< 0.0001**	2.41	3.78
Before or After	-0.87	0.17	-4.98	17	**0.0001**	-1.22	-0.51
**Random effects:**	**s.d.**				**Residual**		
Frog ID	0.48						
Days post exposure/Frog ID	0.88				0.51		

Lechriodus fletcheri

**Fixed effect**	**estimate**	**s.e.**	**t-value**	**d.f.**	***p***	**CI**	
						**2.5%**	**97.5%**
Intercept	5.10	0.76	6.76	5	**< 0.0011**	3.33	6.87
Before or After	-0.84	0.22	-3.73	5	**0.014**	-1.37	-0.31
**Random effects:**	**s.d.**				**Residual**		
Frog ID	7.3×10^{-5}						
Days post exposure/Frog ID	1.65				0.36		

Platyplectrum ornatum

**Fixed effect**	**estimate**	**s.e.**	**t-value**	**d.f.**	***p***	**CI**	
						**2.5%**	**97.5%**
Intercept	5.04	0.19	26.85	2	**0.0014**	4.38	5.70
Before or After	-1.46	0.22	-6.56	2	**0.023**	-2.23	-0.68
**Random effects:**	**s.d.**				**Residual**		
Frog ID	0.10						
Days post exposure/Frog ID	0.10				0.22		

S6 Table. Statistical results from a phylogenetic linear mixed model (implemented in ASReml-R) examining the percent change in infection load after sloughing (log (zoospore equivalents [ZE]+1)) in five species of Australian frog exposed to *Batrachochytrium dendrobatidis* (*Bd*). Fixed effects were the interaction between *days post exposure* and *species*, and random effects were *Frog ID* nested in *Species, days post exposure*, and a variance-covariance matrix created from the phylogeny, to take into account correlated error from repeated measures on the same individual, and phylogenetic non-independence. s.e. = standard error, d.f. = degrees of freedom, bold p-values are significant, λ = phylogenetic signal

**Fixed effects**	**Estimate**	**s.e.**	**F**	**d.f.**	***p***
Intercept	-26.24	14.24	8.40	1, 2.3	0.19
Species: Days post exposure (*Lechriodus fletcheri*)	NA	NA	0.38	4, 2.9	0.81
Species: Days post exposure (*Limnodynastes peronii*)	-4.03	3.25			
Species: Days post exposure (*Limnodynastes tasmaniensis*)	-2.37	3.46			
Species: Days post exposure (*Litoria caerulea*)	-2.03	3.21			
Species: Days post exposure (*Platyplectrum ornatum*)	-4.58	8.32			
Days post exposure	2.48	3.11	0.065	1, 10.5	0.80
**Random effects**	**Variance**	**s.e.**	χ^2	**d.f.**	***p***
Phylogeny	2.53×10^{-5}	5.82×10^{-3}	-7.6×10^{-7}	1	1
Frog ID nested in Species	1.20	355.0	NA	NA	NA
**λ = 8.94×10^{-6}, s.e. = 0.00072**					

S7 Table. Statistical results from linear mixed effects models examining the change in log intermolt interval (IMI) in five species of Australian frog exposed to *Batrachochytrium dendrobatidis* (*Bd*). Fixed effects were the interaction between *group* (control, clinical, infected, uninfected) and *days post exposure*, and *Frog ID* was included as a random factor to take into account correlated error from repeated measures on the same individual. If the interaction between *Group* and *days post exposure* was significant, results of post-hoc comparisons between groups of log IMI across days post exposure are presented beneath model outputs (package = phia, function = testInteraction). s.e. = standard error, s.d. =standard deviation, d.f. = degrees of freedom, CI= confidence intervals, bold p-values are significant

Litoria caerulea

**Fixed effect**	**estimate**	**s.e.**	**t-value**	**d.f.**	***p***	**CI**	
						**2.5%**	**97.5%**
Intercept	1.97	0.016	124.17	121	**< 0.0001**	1.92	1.99
Group (Clinical)	0.089	0.034	2.65	14	**0.019**	1.87×10^{-2}	0.16
Group (Uninfected)	0.006	0.021	0.30	14	0.77	-3.74×10^{-2}	0.05
Days post exposure	0.002	0.0004	4.55	121	**< 0.0001**	9.20×10^{-4}	0.002
Groups (Clinical)*Days post exposure	-0.006	0.0008	-7.20	121	**< 0.0001**	-7.12×10^{-3}	-0.004
Groups (Uninfected)*Days post exposure	0.0008	0.0005	1.74	121	0.084	-9.25×10^{-5}	0.002
**Random effects:**	**s.d.**				**Residual**		
Frog ID	0.033				0.031		

Pairwise comparison of groups (IMI across Days post exposure)

Comparison	Estimate	d.f.	X^2	*p*
Clinical - Control	-0.0056	1	54.15	$\mathbf{3.71 \times 10^{-13}}$
Clinical - Uninfected	-0.0064	1	73.88	$\mathbf{<2.2 \times 10^{-16}}$
Control - Uninfected	-0.0008	1	3.17	0.075

Limnodynastes tasmaniensis

**Fixed effect**	**estimate**	**s.e.**	**t-value**	**d.f.**	***p***	**CI**	
						**2.5%**	**97.5%**
Intercept	1.79	0.025	71.66	160	**< 0.0001**	1.74	1.84
Group (Infected)	0.086	0.033	2.57	8	**0.033**	0.01	0.16
Group (Uninfected)	0.016	0.043	0.38	8	0.71	-0.081	0.11
Days post exposure	-0.0006	0.0005	-0.12	160	0.91	-0.0011	0.001
Groups (Infected)*Days post exposure	-0.003	0.0006	-3.72	160	**0.0003**	-0.004	-0.001
Groups (Uninfected)*Days post exposure	0.001	0.0009	1.21	160	0.23	-0.0006	0.0028
**Random effects:**	**s.d.**				**Residual**		
Frog ID	0.039				0.062		

Pairwise comparison of groups (IMI across Days post exposure)

Comparison	Estimate	d.f.	X^2	*p*
Uninfected - Control	0.0011	1	1.52	0.22
Uninfected - Infected	0.0036	1	19.64	**2.81 x 10^{-5}**
Control - Infected	0.0025	1	14.32	**0.00031**

Limnodynastes peronii

**Fixed effect**	**estimate**	**s.e.**	**t-value**	**d.f.**	***p***	**CI**	
						**2.5%**	**97.5%**
Intercept	1.77	0.026	68.58	179	**< 0.0001**	1.72	1.81
Group (Clinical)	-0.022	0.041	-0.54	6	0.61	-0.12	0.076
Group (Uninfected)	-0.019	0.034	-0.55	6	0.60	-0.10	0.063
Days post exposure	0.0003	0.0006	0.39	179	0.69	-0.001	0.001
Groups (Clinical)*Days post exposure	-0.0018	0.0009	-1.82	179	0.071	-0.0036	0.0011
Groups (Uninfected)*Days post exposure	-0.001	0.0008	-1.33	179	0.18	-0.0027	0.0005
**Random effects:**	**s.d.**				**Residual**		
Frog ID	0.033				0.074		

Lechriodus fletcheri

**Fixed effect**	estimate	s.e.	t-value	d.f.	*p*	CI	
						**2.5%**	**97.5%**
Intercept	1.86	0.011	162.53	78	**< 0.0001**	1.84	1.88
Group (Clinical)	0.019	0.017	1.14	3	0.34	-0.032	0.0069
Group (Infected)	-0.011	0.021	-0.51	3	0.64	-0.075	0.0053
Group (Uninfected)	-0.019	0.034	0.057	3	0.96	-0.071	0.0073
Days post exposure	-0.0009	0.0005	-2.06	78	**0.042**	-0.0018	-7.38x10^{-5}
Groups (Clinical)*Days post exposure	-0.0018	0.0007	-2.51	78	**0.014**	-0.0032	-4.43x10^{-4}
Groups (Infected)*Days post exposure	0.0009	0.0008	1.14	78	0.26	-0.0006	0.00025
Groups (Uninfected)*Days post exposure	0.0007	0.001	0.77	78	0.44	-0.0011	0.00026
**Random effects:**	**s.d.**				**Residual**		
Frog ID	0.0055				0.034		

Pairwise comparison of groups (IMI across Days post exposure)

Comparison	Estimate	d.f.	X^2	*p*
Clinical - Control	-0.0018	1	6.94	**0.042**
Clinical - Infected	-0.0027	1	10.77	**0.0062**
Clinical - Uninfected	-0.0026	1	6.88	**0.042**
Control - Infected	-0.00092	1	1.42	0.70
Control - Uninfected	-0.00074	1	0.65	0.84
Infected - Uninfected	0.00018	1	0.023	0.86

Platyplectrum ornatum

**Fixed effect**	**estimate**	**s.e.**	**t-value**	**d.f.**	***p***	**CI**	
						**2.5%**	**97.5%**
Intercept	1.82	0.036	51.24	33	**< 0.0001**	1.75	1.88
Group (Clinical)	0.076	0.046	1.65	8	0.14	-0.025	0.18
Days post exposure	0.0014	0.0016	0.92	33	0.36	-0.0016	0.004
Groups (Clinical)*Days post exposure	-0.0029	0.0018	-1.63	33	0.11	-0.0064	0.0006
**Random effects:**	**s.d.**				**Residual**		
Frog ID	0.011				0.054		

S8 Table. Examination of the relationship between intermolt interval (IMI) and *Batrachochytrium dendrobatidis* (*Bd*) infection load. A) Statistical results from a phylogenetic linear mixed model (implemented in ASReml-R) examining the change in log IMI with *Bd* load in zoospore equivalents (ZE). Fixed effects were *Bd load* (log[ZE+1]), and random effects were *Frog ID* nested in *Species* and a variance-covariance matrix created from the phylogeny, to take into account correlated error from repeated measures on the same individual, and phylogenetic non-independence. B) Results from a mixed effects model, and corresponding pairwise tests, comparing the relationship between log IMI and log *Bd* load in the three species that increased their sloughing rates when either clinically infected (*Lit. caerulea, Lec. fletcheri*) or infected (*Lim. tasmaniensis*; see S6 Table). s.e. = standard error, d.f. = degrees of freedom, bold p-values are significant.

A) Phylogenetic linear mixed model

**Fixed effects**	**Estimate**	**s.e.**	**F**	**d.f.**	***p***
Intercept	1.90	0.055	1194	1,3.9	**5.04×10^{-6}**
Bd load (log[ZE+1])	-0.016	0.0035	20.79	1,145.6	**1.08×10^{-5}**
**Random effects**	**Variance**	**s.e.**	**χ^2**	**d.f.**	***p***
Phylogeny	1.89	0.006	2.28	1	0.13
Frog ID nested in Species	0.054	3.2×10^{-4}	0.66	1	0.42
**λ = 0.64, s.e. = 0.17**					

B) Mixed model and pairwise tests comparing species that increased sloughing rate

**Fixed effect**	estimate	s.e.	t-value	d.f.	*p*	CI **2.5%**	CI **97.5%**
Intercept	1.87	0.021	-0.38	69	**<0.001**	1.83	1.91
Log *Bd* load	-0.006	0.006	-1.07	69	0.29	-0.018	0.0051
Species (*Lim. tasmaniensis*)	-0.022	0.025	-0.88	10	0.40	-0.075	0.031
Species (*Lit. caerulea*)	0.125	0.026	4.78	10	**0.0007**	0.069	0.18
Log *Bd* load* Species (*Lim. tasmaniensis*)	-0.029	0.010	-2.94	69	**0.0045**	-0.049	-0.010
Log *Bd* load* Species (*Lit. caerulea*)	-0.0059	0.0085	-0.69	69	0.49	-0.022	-0.010
**Random effects:**	**s.d.**				**Residual**		
Frog ID	1.04×10^{-6}				0.053		

Pairwise comparison of log IMI across log *Bd load*

Comparison	Estimate	d.f.	X^2	*p*
Lec. fletcheri - Lim. tasmaniensis	0.029	1	9.29	**0.0069**
Lec. fletcheri - Lit. caerulea	0.0059	1	0.52	0.47
Lim. tasmaniensis - Lit. caerulea	-0.024	1	5.94	**0.030**
